# Spatiotemporal distribution of human brucellosis in Inner Mongolia, China, in 2010–2015, and influencing factors

**DOI:** 10.1038/s41598-021-03723-9

**Published:** 2021-12-20

**Authors:** Danyan Liang, Dan Liu, Min Yang, Xuemei Wang, Yunpeng Li, Weidong Guo, Maolin Du, Wenrui Wang, Mingming Xue, Jing Wu, Buyun Cui, Shaohua Yin, Ruiqi Wang, Shiyuan Li

**Affiliations:** 1 School of Public Health, Inner Mongolia Medical University, Hohhot, Inner Mongolia China; 2 Inner Mongolia People’s Hospital, Hohhot, China; 3 Department of Medicine, Hetao College, Bayan Nur, China; 4 Inner Mongolia Ecology and Agrometeorology Center, Hohhot, China; 5 Inner Mongolia Center for Disease Control and Prevention, Hohhot, China; 6 School of Basic Medicine, Inner Mongolia Medical University, Hohhot, China; 7 National Center for Chronic and Non-Communicable Disease Control and Prevention, Chinese Center for Disease Control and Prevention, Beijing, China; 8 National Institute for Communicable Disease Control and Prevention, Chinese Center for Disease Control and Prevention, Beijing, China

**Keywords:** Public health, Risk factors

## Abstract

Human brucellosis is caused by Brucella species and remains a major burden in both human and domesticated animal populations, especially in Inner Mongolia, China. The aims of this study were to analyze the spatiotemporal trends in human brucellosis in Inner Mongolia during 2010 to 2015, to explore the factors affecting the incidence of brucellosis. The results showed that the annual incidence was 29.68–77.67 per 100,000, and peaked from March to June. The majority of human brucellosis was male farmers and herdsmen, aged 40–59 years. The high-risk areas were mainly Xilin Gol League and Hulunbeier City. The incidence of human brucellosis in Inner Mongolia decreased during 2010 to 2015, although the middle and eastern regions were still high-risk areas. The regions with larger number of sheep and cattle, lower GDP per capita, less number of hospital beds, higher wind speed, lower mean temperature more likely to become high-risk areas of human brucellosis.

## Introduction

Human brucellosis is caused by *Brucella* species and is a zoonotic infectious disease. According to the World Health Organization, more than 500,000 new cases of human brucellosis occur globally each year. Human brucellosis is prevalent in low and middle-income regions, such as the Mediterranean, central Asia, the Middle East, Latin America, sub-Saharan Africa, and the Balkans^1–6^. Human brucellosis is listed as one of the statutory notifiable infectious diseases by the World Organization for Animal Health^7^. Throughout the twentieth century, the Chinese Government undertook a series of targeted control measures, which were generally effective at controlling human brucellosis outbreaks^8,9^. In the twenty-first century, there was a human brucellosis resurgence and the disease expanded its range into new areas^10,11^. To date, human brucellosis outbreaks have been reported in 28 areas of China, including Inner Mongolia, Jilin, and Heilongjiang^12^. During 2004–2016, a total of 448,479 cases of brucellosis were confirmed in China, depicting an amplified trend for the epidemic across all provinces^13^. Inner Mongolia is one of the most seriously affected regions in China and considered a focal area for study of human brucellosis. During 1999–2008, 43,623 human brucellosis cases were reported in Inner Mongolia^14^. In 2005–2010, Inner Mongolia accounted for 33.2–68.3% of the country’s total human brucellosis burden^12^. In 2011, 20,845 cases of human brucellosis were reported in Inner Mongolia^11^, accounting for 45.8% of all cases in China in 2013^15^. From 2010 to 2014, the total seropositive and incidence rate of human brucellosis in Inner Mongolia was 35.91‰ and 18.25‰, respectively^16^.

The main purposes of this study were to describe the epidemiology of human brucellosis in Inner Mongolia during 2010–2015, to investigate the spatiotemporal pattern of human brucellosis and the association with risk factors of the disease.

## Materials and methods

### Study areas

Inner Mongolia is located on the northern border of China, and covers about 118.3 million km^2^. Inner Mongolia has 12 municipal city level administrative units, and a total of 101 counties.

### Data source

Human brucellosis case data from January 1, 2010, to December 31, 2015 were obtained from the Centers for Disease Control and Prevention in Inner Mongolia. Ultimately, 76,907 patients were included in this study. Monthly meteorological data were obtained from the Inner Mongolia Meteorological Bureau. Socioeconomic data for each year were obtained from the Inner Mongolia Autonomous Region Statistical Yearbooks. Gross Domestic Product (GDP), bed numbers, the number of cattle and sheep were calculated on a monthly basis, and all data were standardized (z-score model: $z = \frac{{X - \overline{X} }}{s}$).

### Statement

Our study was reviewed by the Ethics Committee of Inner Mongolia Medical University. All laboratory tests were in accordance with ISO 15189 guidelines. The diagnosis of brucellosis was based on the diagnostic criteria and treatment principles of brucellosis proposed by the Ministry of Health of the People's Republic of China. The collection of brucellosis data was in accordance with the provisions of the law of the People's Republic of China on the prevention and control of infectious diseases. The collection of data was approved by the Inner Mongolia Center for Disease Control and Prevention and the patient's name was hidden to protect the patient's privacy. Data collection was obtained with the informed consent of all participants, or if participants are under 18, from a parent and/or legal guardian.

### Statistical methods

R 3.3.2 was used for data organization. We used the Open BUGS 3.2.3 software for the Bayesian model. ArcGIS 10.2 software was used for the spatial autocorrelation test, and to map the spatial distribution of human brucellosis. The Econometric Models for Spatial Panel Data (‘splm’) package in R^17^ was used for the spatial panel analysis (https://www.r-project.org/).

### Bayesian spatiotemporal model

Familiar Bayesian Spatial–temporal Model (FBM) used in this study is a type of Bayesian spatiotemporal model that was established by Li et al.^18^. We used the Poisson distribution as the joining function for the FBM model: $y_{it} \sim Poisson\;(n_{it} u_{it} )$. Specifically, we let $y_{it}$, $n_{it}$, and $u_{it}$ represent the number of newly diagnosed cases, the permanent population at the end of a year, and morbidity rate per county per year, respectively, in counties $i$ (= 1, …, 101) at time point $t$ (= 1, 2, …, 5).

$$\log (u_{it} ) = \alpha + s_{i} + b{}_{0}t^{*} + \upsilon_{t} + b_{1i} t^{*} + \varepsilon_{it} .$$

Under this model, the observed space–time variability in brucellosis risk is decomposed into the following components. $\alpha$ represents the overall log risk of brucellosis in Inner Mongolia during the study period. The spatial term $s_{i}$, common across the five observation years, describes the distribution of the risks of human brucellosis. $b_{0} t^{*} + \upsilon_{t}$ describes the overall temporal trend (common across all counties). The overall temporal trend is specified represented as a linear trend ($b_{0} t$) with additional Gaussian noise ($\upsilon_{t}$), which allows for nonlinearity in the overall trend pattern. $t^{*} = t - 3$ (centering at the mid observation period). The combination of the common spatial pattern and the common time trend represents the stable component of disease risk. The term $b_{1i} t^{*}$ allows each county to have its own trend, which captures any additional variability in risk for each county over and above the spatial and temporal trend components. While $b_{0}$ represents the overall rate of change in risk, $b_{1i}$ measures the departure from $b_{0}$ for each county. For example, a negative estimate of $b_{1i}$ would suggest a slower increase (or even a decline) in risk over time for that county. The last term $\varepsilon_{it}$ captures additional variability in the data not explained by other model components, which is a random error term for the spatiotemporal interaction.

For, the spatial weight matrix of this study, we used the space adjacency matrix $W_{101 \times 101}$, if the regions $i$ and $j$ are adjacent (those counties that shared a common border), then $W_{ij} = 1$, whereas if they are not, $W_{ij} = 0$.

### Spatial autocorrelation analysis

Moran’s *I* index is a commonly used statistic for detecting spatial clustering. Moran’s *I* > 0 indicates that the regional variables show objects closer together are similar to the objects surrounding it. Moran’s *I* < 0 indicates that objects closer together are dissimilar to the objects surrounding it. Moran’s *I* = 0 indicates that the regionalized variables are randomly distributed in space.

### Spatial panel data model

Spatial panel data model was used to determine factors affecting the temporal and spatial distributions of human brucellosis. The parameters in the spatial panel data model can be estimated with the maximum likelihood estimates. The general form of the spatial panel data model is:

$$y_{it} = \rho W_{i \times j} y_{it} + \beta x + u$$

$$u = \lambda W_{i \times j} u + \varepsilon$$

where $x$ and $y$ are the observed variables, $i$ and $j$ are the spatial position, $t$ represents time in months. $\rho W_{i \times j} y_{it}$ is the dependent variable space lag term, and $\rho$ is the spatial autocorrelation coefficient used to measure the effect of $W_{i \times j} y_{it}$ on $y_{it}$. $W_{i \times j}$ is the spatial weight matrix, and $\beta$ is the regression coefficient. $u$ is the perturbation term, $\lambda$ is the spatial autocorrelation coefficient, and $\varepsilon$ is the random effect. When $\rho = 0$, the model is a spatial error model, and the spatial autocorrelation is the spatial correlation because of the spatial clustering of the dependent variables.

## Results

### Descriptive analysis

**Figure 1:**
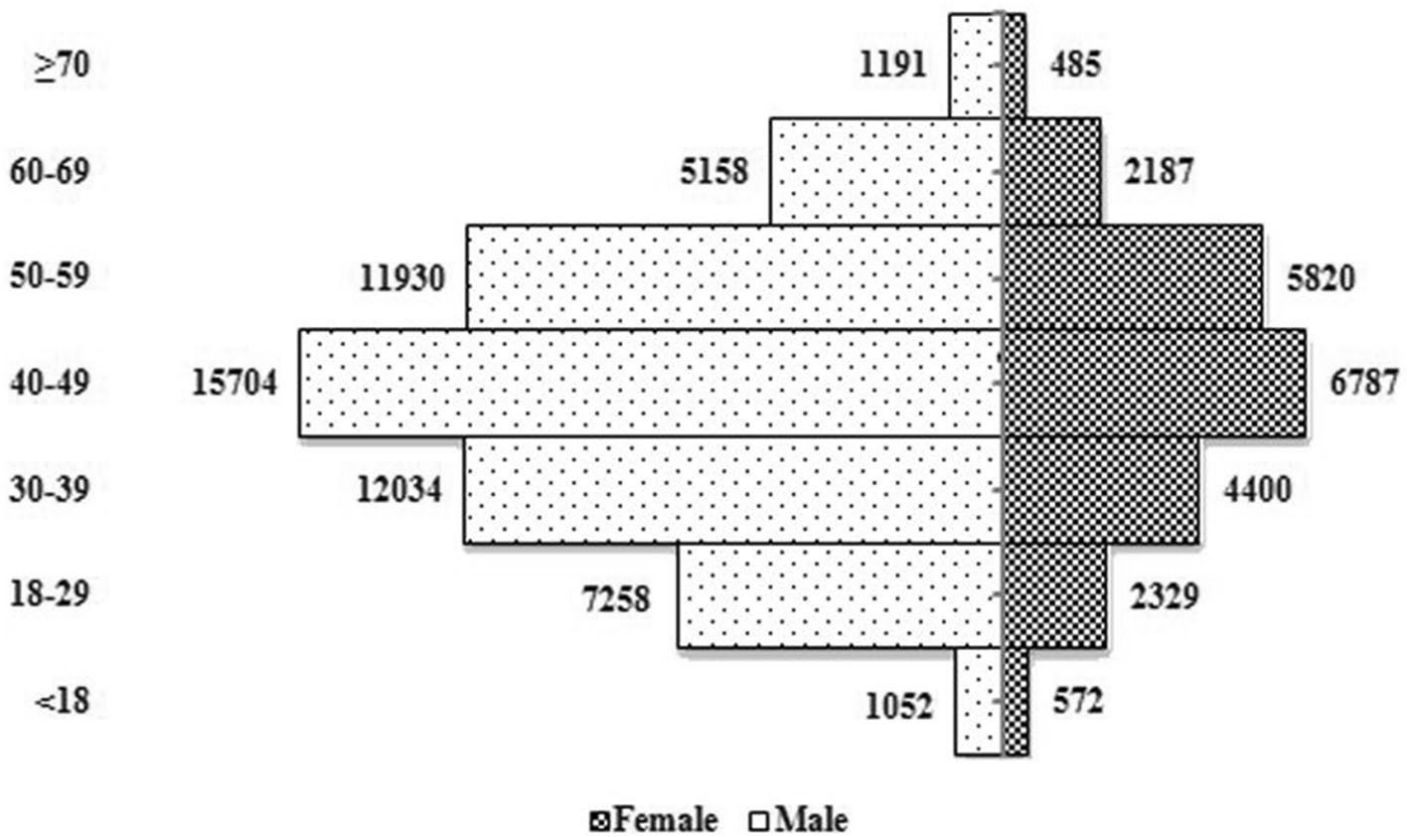
Number of newly diagnosed brucellosis cases for male and female patients in different age groups, in Inner Mongolia, 2010–2015.

In 2010–2015, the prevalence rate of male with brucellosis reached 69.92% (Fig. 1). The highest prevalence in the 40–49 age group was 28.69%, followed by those aged 50–59 years (26.33%) and those aged 30–39 years (17.50%).

**Figure 2:**
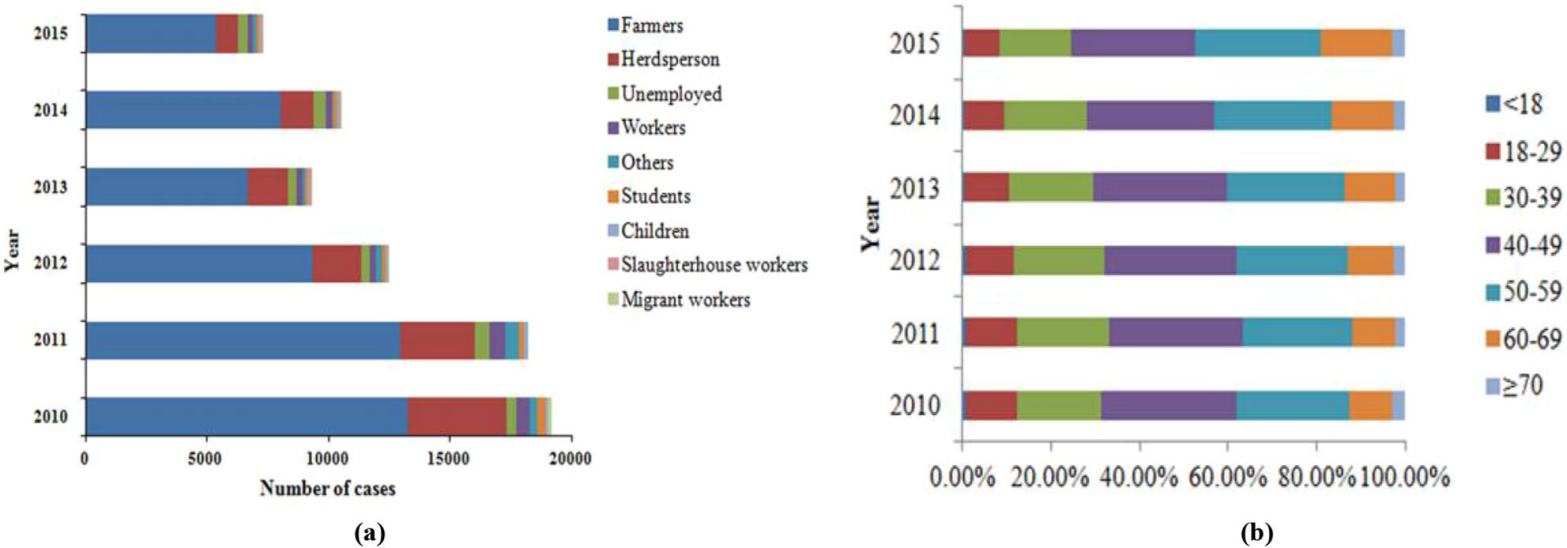
Brucellosis cases for different groups in different age groups, in Inner Mongolia, 2010–2015. (**a**) Annual brucellosis cases for different groups in Inner Mongolia, 2010–2015. (**b**) Age composition of farmer patients, in Inner Mongolia, 2010–2015.

The majority of cases (72.3%) were farmers and 17.0% were herdsperson (Fig. 2a). In the age groups of farmers, the proportion of 40–49 years shows a downward trend year by year, accounted for 30.31%, 30.20%, 29.99%, 29.85%, 28.95% and 28.11%, during 2010–2015 (Fig. 2b).

**Figure 3:**
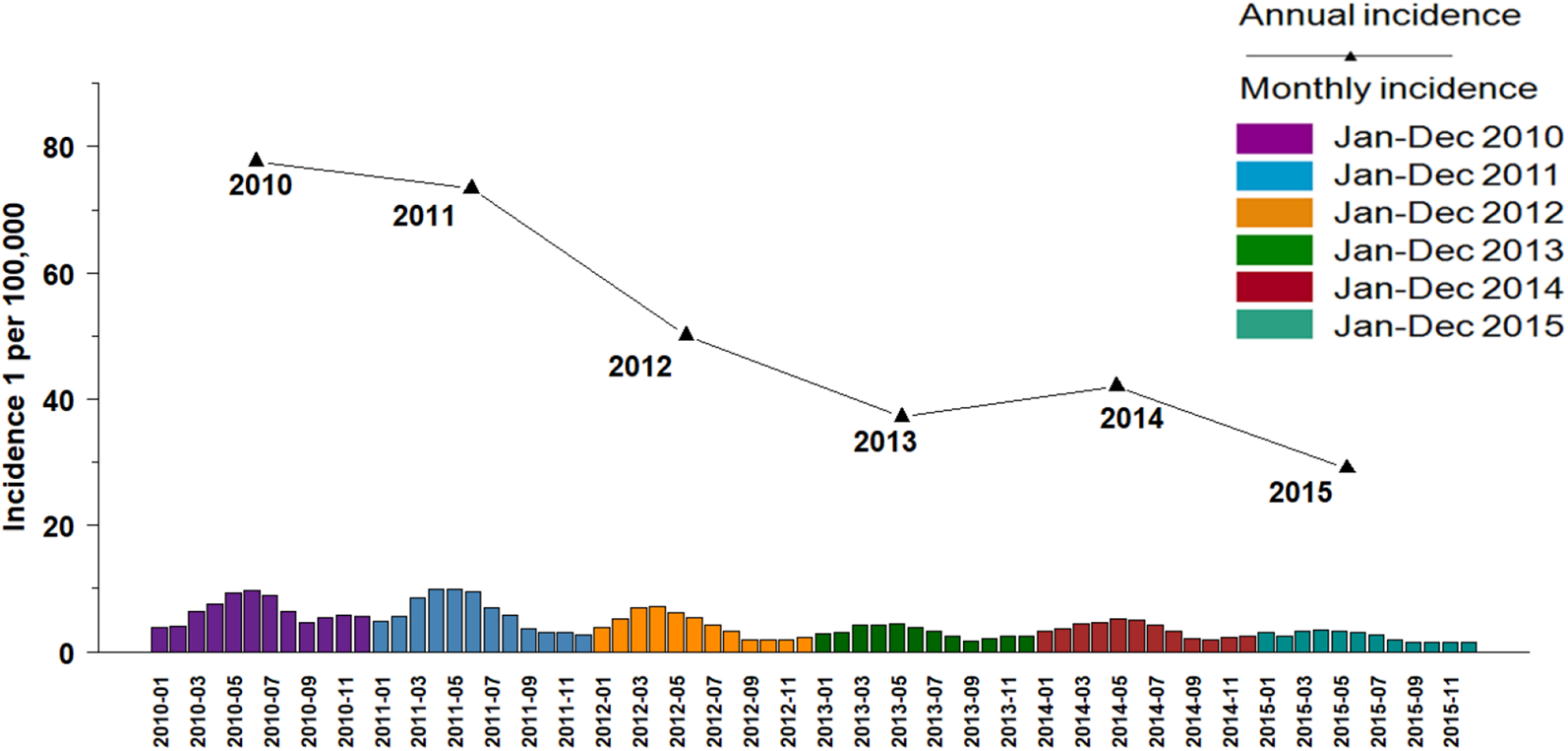
Temporal distribution of brucellosis in Inner Mongolia, 2010–2015.

Over 2010–2015 study period, the incidence of human brucellosis from March to June was higher than in other months, accounted for 47.07% (Fig. 3).

### Spatiotemporal distribution

**Figure 4:**
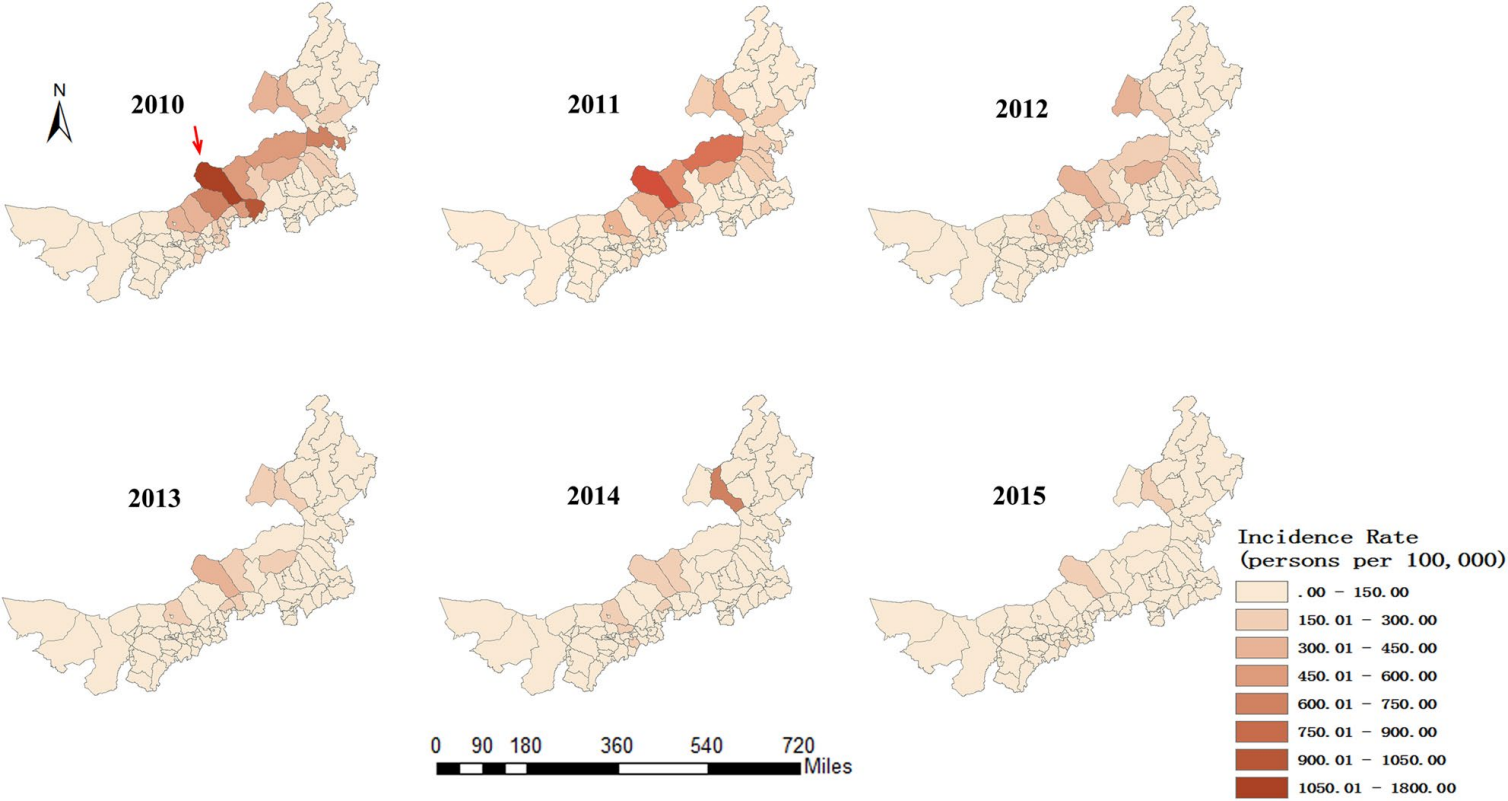
Spatial distribution of the annual incidence rates of brucellosis in 101 counties in Inner Mongolia, 2010–2015.

The annual incidence of human brucellosis per 100,000 was 77.67 in 2010, 72.11 in 2011, 49.51 in 2012, 36.62 in 2013, 41.56 in 2014, and 29.68 in 2015. Sunitezuo Banner (Pointed by the red arrow), one of the areas with the highest incidence of the human brucellosis, where the incidence rate decreased from 1720.79/100,000 in 2010 to 187.60/100,000 in 2015. The incidence of each county during 2010–2015 shown in Fig. 4.

### Spatial autocorrelation analysis

We analyzed the spatial autocorrelation of human brucellosis incidence in 101 counties and of Inner Mongolia (as shown in Table 1). 2010 to 2013, Moran’s I was > 0 (*P* < 0.001), indicating a spatial autocorrelation.

**Table 1:** Spatial autocorrelation analysis.

Year	Moran’s *I*	*Z* score	*P*
2015	0.061	1.286	0.199
2014	0.056	1.457	0.145
2013	0.172	3.326	0.001
2012	0.203	3.800	< 0.001
2011	0.182	3.542	< 0.001
2010	0.153	3.217	0.001

### Bayesian spatial model analysis

**Figure 5:**
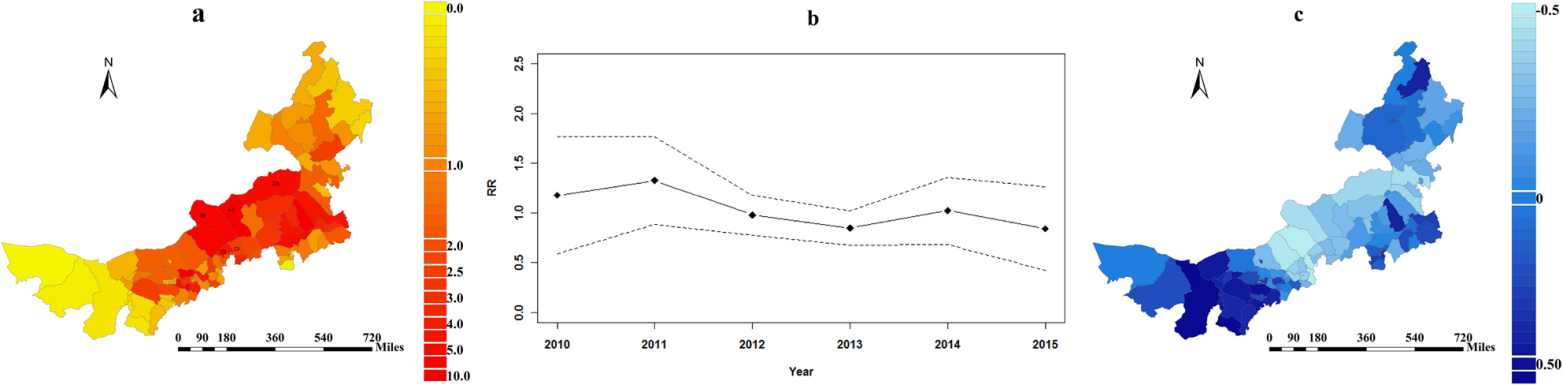
Temporal and spatial trends of brucellosis in Inner Mongolia, 2010–2015. (**a**) The common spatial component (the posterior mean of the spatial relative risk, exp [$s_{i}$]). (**b**) The overall time trend with 95% CI (the posterior mean of the temporal relative risks, exp [$b_{0} t^{*} + \upsilon_{t}$]. (**c**) The departure of the local trends from the overall trend (the posterior mean of *b*_*1i*_).

The spatial risk pattern for human brucellosis in the counties of Inner Mongolia is shown in Fig. 5a. The high-risk areas included Abaga Banner, Sunitezuo Banner, Dongwuzhumuqin Banner, Zhengxiangbai Banner, and Xianghuang Banner (Pointed by the black square). Figure 5b was the common time trend of the incidence of brucellosis in Inner Mongolia. The temporal trend of brucellosis in Inner Mongolia fluctuated greatly, and the overall trend of the relative risk (*RR*) decreased from 1.134 (95% CI 1.092–1.176) in 2010 to 0.797 (95% CI 0.767–0.826) in 2015. The extent to which the risk of disease in each county deviates from the overall risk is shown in Fig. 5c. Compared with the overall decreasing trend, human brucellosis in the east and west tended to decrease more rapidly over time. A panel with the summation of $s_{i} + b_{1i} t$ showed that the highest total risk is the Xilin Gol League (Supplemental Fig. 1).

The spatial risk ($s_{i}$) and the temporal trend ($b_{1i}$) were used for classification. The risk of human brucellosis was divided into three levels: level A, *RR* > 2, hotspot; level B, 0.5 < *RR* ≤ 2, neither hotspot nor coldspot; level C, *RR* ≤ 0.5, coldspot. With $b_{1i}$ the effect of the time and space interaction was divided into three levels: level 1, $b_{1i}$ ≥ 0.16, the reduction in the risk of disease is faster than the overall trend; level 2, − 0.18 ≤ $b_{1i}$ < 0.16, the reduction in the disease risk is equivalent to the mean level; level 3, $b_{1i}$ < − 0.18, the reduction in the disease risk decreases more slowly than the mean trend (Supplemental Table 1).

### Spatial panel data model analysis

First, we performed the Hausman test, with the result χ^2^ = 33.55 (*P* < 0.001), suggesting that there was no random effect in the spatial panel model. The Lagrange multiplier (LM) = 313.20 (*P* < 0.001), suggesting that the spatial autocorrelation mainly occurred in the error term, when the spatial panel error model was used to analyze the factors related the incidence of disease. The results showed that there was a negative correlation between human brucellosis incidence and GDP [*β* = − 0.087, 95% CI (− 0.122, − 0.052)] and the number of hospital beds [*β* = − 0.116, 95% CI (− 0.149, − 0.083)]. There was a positive correlation between human brucellosis incidence and the number of cattle [*β* = 0.073, 95% CI (0.040, 0.106)] and the number of sheep [*β* = 0.107, 95% CI (0.078, 0.136)]. There was a negative correlation between human brucellosis incidence and temperature [*β* = − 0.462, 95% CI (− 0.613, − 0.311)], whereas there was a positive correlation with wind speed [*β* = 0.181, 95% CI (0.148, 0.214)] (Table 2).

**Table 2:** Factors influencing the incidence of brucellosis.

Variable	*β*	Standard error	*t*	*P*	*β* 95% CI	
					LL	UL
Number of cattle	0.073	0.017	4.264	< 0.001	0.040	0.106
Number of sheep	0.107	0.015	7.143	< 0.001	0.078	0.136
Gross Domestic Product	− 0.087	0.018	4.806	< 0.001	− 0.122	− 0.052
Number of hospital beds	− 0.116	0.017	6.475	< 0.001	− 0.149	− 0.083
Mean temperature	− 0.462	0.077	5.969	< 0.001	− 0.613	− 0.311
Mean wind speed	0.181	0.017	10.532	< 0.001	0.148	0.214
Relative humidity	− 0.019	0.032	0.592	0.554	− 0.082	0.044
Average rainfall	− 0.017	0.027	0.637	0.524	− 0.070	0.036
Average sunshine hours	− 0.032	0.028	1.111	0.267	− 0.087	0.023
Constant	0.394	0.013	29.608	< 0.001	0.369	0.419

## Discussion

The descriptive analysis showed that male in Inner Mongolia accounted for 70% of diseased individuals, consistent with the other results^14^. This is probably a reflection of the occupational exposure of males to feed and slaughter of animals, whereas females are less frequently exposed to livestock in their domestic duties. Our results showed that individuals age 40–59 account for the highest percentage of cases across all age groups. This is due to they play an important role in farm work of the family and increased their exposure to *Brucella*. These findings on the age and gender distribution of human brucellosis were very similar to those of the whole country of China^10^. Owing to the downwards temporal trend in cases, there was insufficient data in 2014 and 2015 to detect any spatial clustering.

Human brucellosis cases were predominantly farmers or herdsmen because those agricultural workers are involved in animal slaughter, delivery of lambs, the sale of animal products, and other high-risk activities, increasing their risk of infection. This result is consistent with the findings from Hebei and Shanxi in China^19,20^. On the one hand, farmers often slaughter livestock for their own consumption without meat inspection. On the other hand, compared to slaughterhouse experts, local farmers did not wear any protective measures, resulting in a high incidence of human brucellosis infection.

The temporal distribution analysis showed that human brucellosis in Inner Mongolia reported mainly from March to June, i.e. in spring and summer. This is because human brucellosis is predominantly transmitted by infected pregnant animals. Parturition or abortion in winter and spring increases the prevalence of *Brucella* bacteria in the environment. However, the peak period of human brucellosis does not exactly match the production season of animals. There are probably two main reasons: Firstly, the mean incubation period for human brucellosis in the human body is 2–4 weeks^21^. Secondly, study showed that 24% of patients delayed treatment^15^, therefore disease reported focuses on spring and summer could be because of this lag effect.

The distribution of human brucellosis was mainly concentrated in Xilin Gol League and Hulunbeier in central and eastern Inner Mongolia, which is consistent with previous reports^14^. Consistent with Tongliao^22^, Xilin Gol League is the most important livestock husbandry center in China and has vast grasslands, which may provide a ‘hotbed’ for the spread of human brucellosis. Furthermore, the livestock trade in this region is extensive, which increases the chance of livestock infection. In our study, between 2010 and 2015, human brucellosis was effectively controlled, and the incidence was significantly lower than in a 2004–2010 study^23^. In 2012, government departments in the Inner Mongolia instituted a new disease prevention and control plan, and developed a series of diagnostic and treatment programs, together with publicity and education programs^22,11^, which have been shown to be effective^24,25^.

We should focus on the prevalence of human brucellosis in humans and the monitoring of livestock’s infection will be beneficial for prevention and control human brucellosis^26^. In our study sheep and cattle are the main hosts of *Brucella* and mainly transmitted from its animal reservoirs. At present, many inconsistent findings about animal reservoir of human brucellosis have appeared. One of other important sources for human brucellosis is cattle that are very susceptible to *Brucella* and human cases due to *Brucella abortus* are commonly sporadic reported^9^. Both sheep and cattle have potential to transmit the disease to humans^27^. A study showed that 90% of human brucellosis was small-ruminant derived in Mongolia, a neighboring country of Inner Mongolia^28^.

In our study there was a negative correlation between local GDP and incidence of human brucellosis. Previous research has highlighted that human brucellosis is more severe in counties with low GDP^29^. High-income countries can implement better disease prevention and control measures, because of greater financial support and material resources. We represented the number of hospital beds as proxy variable of the medical level of the area. This is consistent with the previously reported that low levels of medical services and shortages of medical resources may lead to higher prevalence of zoonotic diseases^30^. In areas with poor medical care, misdiagnosis and underreporting may be more common. Except for traditional control measures, previous studies have pointed out that more attention should be paid to improving medical care to improve control effectiveness, especially in rural areas^31^. Therefore, we should take care to prevent the outbreak of human brucellosis in low-medical care areas.

Meteorological and environmental models of human brucellosis are relatively rare, because human brucellosis is not as sensitive to climate as other infectious diseases^32,33^. However, the changing environment may lead to drought and degradation of pastures, which will further increase the sensitivity of animals with lower drug resistance to disease^34^, and affect the activity of the host and the survival of *Brucella*. Consistent with other study^35^, there was a negative correlation between human brucellosis incidence and temperature. The climate in winter and spring may affect the normal breeding time of livestock, on the other hand, it may increase the chance of close contact between humans and fauna^36^. We believe that temperature affects not only *Brucella* spp. survivors, but also human-animal interactions such as keeping animals captive in cold winters. On the other hand, in winter and spring, cattle and sheep were in the pregnancy, abortion and production will release large amounts of Brucella in the surrounding environment, causing human infection. Studies have shown that *Brucella* can survive for several months in low temperatures, high humidity, and less sunshine in the winter^37,38^. Additionally, Human brucellosis is capable of being transmitted by fomites^39^. Higher wind speeds facilitate the greater spread of pollutants carrying *Brucella*, increasing transmission between livestock populations, further increasing the risk to humans. Moreover, herdsmen tend to raise animals at home rather than grazing in high wind speeds weather, which also increases the risk of human becoming infected with human brucellosis.

## Conclusion

During 2010–2015, the overall incidence of human brucellosis in Inner Mongolia decreased. The middle and eastern regions, such as Xilin Gol League and Hulunbeier were high-risk areas. The areas which are mainly feeding sheep and cattle and have distinguishing characteristic features with low GDP and low level of medical care have high risk of human brucellosis. Among the meteorological factors affecting the outbreak of human brucellosis, the possibility of suffering from human brucellosis was negatively correlated with the average temperature, and conversely, positively correlated with the average wind speed. The majority of human brucellosis were in males, and in those aged 40–59 years; by occupation, farmers and herdsmen were the most frequently affected. Therefore, targeted health education campaigns are needed to improve knowledge and awareness in these populations.

## Supplementary Information

Supplementary Information.
