# Supplementary material for: Spatiotemporal distribution of human brucellosis in Inner Mongolia, China, in 2010–2015, and influencing factors
Source: Sci Rep. 2021 Dec 20;11:24213. doi: 10.1038/s41598-021-03723-9 (PMC8688419; doi:10.1038/s41598-021-03723-9)

**Spatiotemporal distribution of human brucellosis in Inner Mongolia, China, in 2010–2015, and influencing factors**

Danyan Liang^ab#^, Dan Liu^ac#^, Min Yang^a#^, Xuemei Wang^a*^, Yunpeng Li^d^, Weidong Guo^e^, Maolin Du^a^, Wenrui Wang^e^, Mingming Xue^f^, Jing Wu^g^, Buyun Cui^h^, Shaohua Yin^a^, Ruiqi Wang^a^, Shiyuan Li^a^

^a^School of Public Health, Inner Mongolia Medical University, Hohhot, China

^b^Inner Mongolia People's Hospital, Hohhot, China

^c^Department of Medicine, Hetao College, Bayan Nur, China

^d^Inner Mongolia Ecology and Agrometeorology Center, Hohhot, China

^e^Inner Mongolia Center for Disease Control and Prevention, Hohhot, China

^f^School of Basic Medicine, Inner Mongolia Medical University, Hohhot, China

^g^National Center for Chronic and Non-Communicable Disease Control and Prevention, Chinese Center for Disease Control and Prevention, Beijing, China

^h^National Institute for Communicable Disease Control and Prevention, Chinese Center for Disease Control and Prevention, Beijing, China

*Correspondence: Xuemei Wang, School of Public Health, Inner Mongolia Medical University, Hohhot, Inner Mongolia, China. *E-mail address*:wangxm_zsu@163.com

^#^These authors contributed equally to this work.

| Supplemental Table 1 classification of brucrllosis risk for 101 counties in Inner Mongolia, in 2010-2015 | | | | | | | | | |  |  |
| --- | --- | --- | --- | --- | --- | --- | --- | --- | --- | --- | --- |
|  | Risk reduction trend is fast | |  | | Risk reduction trend is slow | |  | | Not differing from the common trend | | |
|  | districts | counts |  | | districts | counts |  | | districts | | counts |
| Hotpots |  | 0 |  | | Qingshuihe County, Duolun County, Xilin Gol League, Zhalute Banner, Huolinguole City, | 23 |  | | Xinbaerhuzuo Banner, Kulun Banner | | 2 |
|  |  |  |  | Zhalantun City, Xinbaerhuyou Banner, Suniteyou Banner, Wulanhaote City, Zhalaite Banner, Shangdu County, Keerqinyouyiqian Banner, Taipusi Banner, | |  |  |  | | |  |
|  |  |  |  | Xiwuzhumuqin Banner, Tuquan County, Zhenglan Banner, | |  |  |  | | |  |
|  |  |  |  | Keerqinyouyizhong Banner, Dongwuzhumuqin Banner, | |  |  |  | | |  |
|  |  |  |  | Zhengxiangbai Banner, Xianghuang Banner, Huade County, | |  |  |  | | |  |
|  |  |  |  | Abaga Banner, Sunitezuo Banner, | |  |  |  | | |  |
| Coldspots | Alashanzuo Banner, Wuda District, | 17 |  | | Huimin District, | 3 |  | | Jiuyuan District, | | 5 |
|  | Etuokeqian Banner, |  |  | Kundulun District, | |  |  | Donghe District, | | |  |
|  | Hainan District, Etuoke Banner, |  |  | Jining District | |  |  | Ningcheng County, | | |  |
|  | Haibowan District, Genhe City, |  |  |  | |  |  | Qingshan District, | | |  |
|  | Alashanyou Banner, Ejina Banner, |  |  |  | |  |  | Baiyun Mineral District | | |  |
|  | Yijinhuoluo Banner, Wushen Banner, |  |  |  | |  |  |  | | |  |
|  | Wulatehou Banner, Dengkou County, |  |  |  | |  |  |  | | |  |
|  | Hangjin Banner, Zhungeer Banner, |  |  |  | |  |  |  | | |  |
|  | Hangjinhou Banner,  Dongsheng District |  |  |  | |  |  |  | | |  |
| Neither hot nor coldspots | Dalate Banner, | 8 |  | | Wengniute Banner, Arong Banner, Saihan District, | 23 |  | | Keerqin District, Songshan District, | | 20 |
|  | Wuyuan County, |  |  | Balinyou Banner, Aohan Banner, Balinzuo Banner, | |  |  | Shiguai District, Keerqinzuoyihou Banner, | | |  |
|  | Linhe District, |  |  | Molidawadawoer National Autonomous Banner, | |  |  | Keerqinzuoyizhong Banner, Guyang County | | |  |
|  | Hongshan District, |  |  | Wuchuan County, Keshiketeng Banner, Xinghe County | |  |  | Tumotezuo Banner, Hailaer District, | | |  |
|  | Yuanbaoshan District, |  |  | Elunchun National Autonomous Banner, Siziwang Banner, | |  |  | Yuquan District, Naiman Banner, Yakeshi City, | | |  |
|  | Wulateqian Banner, |  |  | Xincheng District, Aershan City, Manzhouli City, | |  |  | Tumoteyou Banner, Kailu County, | | |  |
|  | Kalaqin Banner, |  |  | Fengzhen City, Daerhanmaomingan Union Banner, | |  |  | Wulatezhong Banner, Tuoketuo County, | | |  |
|  | Alukeerqin Banner |  |  | Linxi County, Chahaeryouyizhong Banner, | |  |  | Chenbaerhu Banner, Helingeer County, | | |  |
|  |  |  |  | Zhuozi County, Chahaeryouyiqian Banner, | |  |  | Ewenke National Autonomous Banner, | | |  |
|  |  |  |  | Chahaeryouyihou Banner, Erlianhaote City, | |  |  | Liangcheng County, Eerguna City, | | |  |
|  |  |  |  |  | |  |  |  | | |  |


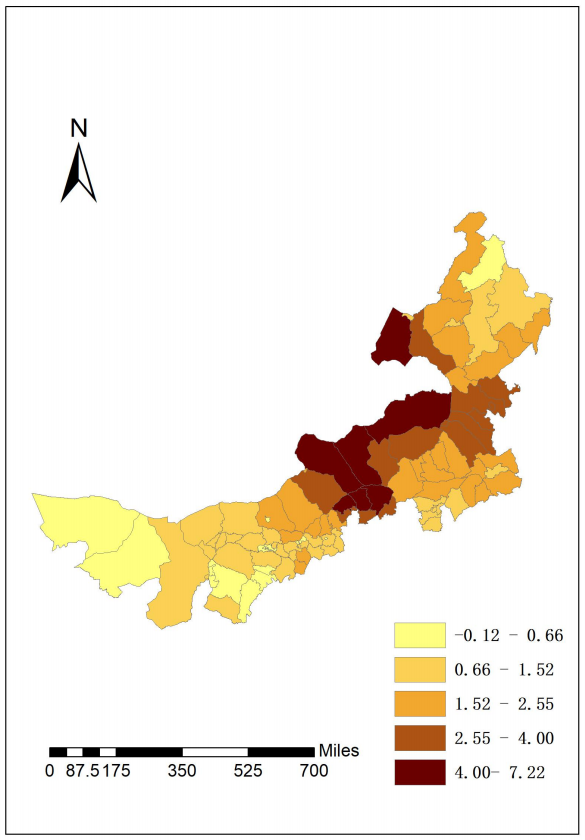

Supplement: Supplementary file 1 — Supplementary Information. [file 41598_2021_3723_MOESM1_ESM.docx]
